# Reliability and type of consumer health documents on the World Wide Web: an annotation study

**DOI:** 10.1186/2041-1480-2-S3-S5

**Published:** 2011-07-14

**Authors:** Melanie J Martin

**Affiliations:** 1 Computer Science Department, California State University, Stanislaus, One University Circle, Turlock California, 95382, USA

## Abstract

**Background:**

In this paper we present a detailed scheme for annotating medical web pages designed for health care consumers. The annotation is along two axes: first, by reliability (the extent to which the medical information on the page can be trusted), second, by the type of page (patient leaflet, commercial, link, medical article, testimonial, or support).

**Results:**

We analyze inter-rater agreement among three judges for each axis. Inter-rater agreement was moderate (0.77 accuracy, 0.62 F-measure, 0.49 Kappa) on the page reliability axis and good (0.81 accuracy, 0.72 F-measure, 0.73 Kappa) along the page type axis.

**Conclusions:**

We have shown promising results in this study that appropriate classes of pages can be developed and used by human annotators to annotate web pages with reasonable to good agreement.

**Availability:**

No.

## Background

With the explosive growth of the World Wide Web has come, not just an explosion of information, but also the explosion of false, misleading and unsupported information. At the same time, the web is increasingly used for tasks where information quality and reliability are vital, from legal and medical research by both professionals and lay people, to fact checking by journalists and research by government policy makers.

In particular, there has been a proliferation of web pages in the medical domain for health care consumers. At the first sign of illness or injury more and more people go to the web before consulting medical professionals. The quality, or reliability, of the information on consumer medical web pages has been of concern for some time to medical professionals and policy makers. For example, in 1997, Impicciatore et al. [1] sampled web documents relating to fever in children and found the quality of the information provided to be very low. In 2002, Eysenbach et al. [2] conducted a review of studies assessing the quality of consumer health information on the web. Of the 79 studies meeting their inclusion criteria (essentially appropriate scope and quantitative analysis), they found that 70% of the studies concluded that reliability of medical information on the Web is a problem. More recent studies have continued to find problems with the reliability of consumer medical information on the Web (e.g. [3]).

Our goal is to create a system that can automatically measure the reliability of web pages in the medical domain [4]. More specifically, given a web page resulting from a user query on a medical topic, we would like to automatically provide an estimate of the extent to which the information on the page can be trusted. In order to make use of supervised natural language processing and machine learning algorithms to create such a system, and to ultimately evaluate the performance of the system, it is necessary to have human annotated data.

The first step in an annotation task is to define the classes we wish to annotate. These definitions need to be meaningful and sufficiently clear so that human annotators can agree. To develop and clarify the definitions used in this work we began by surveying the significant body of literature from librarians, scholars, and information scientists on the quality (reliability) of print, and more recently, web information (for example, see [5], [6]). The question arose whether the surveyed work would translate to the consumer medical domain on the Web. To address this question, Fallis and Frické [7] empirically tested several proposed indicators and found that the standard indicators of quality for print media could not be directly translated to consumer medical information on the Web. Thus the development of our definitions builds on the standard literature and incorporates aspects unique to the Web and aspects specific to the consumer medical domain.

An early distinction we made is between the quality (reliability) and credibility (e.g. [8]), which is based on the user’s view of the information. Here we are interested in the quality or trustworthiness of the information itself. It is important to note the varied uses of the term “reliability” in the computer and information sciences. In the current context we use it to refer to an intrinsic property of a web page: essentially the trustworthiness of the information it contains. This sense of reliability is distinct from its meaning in measurement theory as an indicator of repeatability.

In this paper we report results of an annotation study of medical web pages designed for health care consumers. Three humans annotated two corpora of web pages along two axes. The first axis is the reliability of the information contained in the page. The second axis is the type, or kind, of page. In our methods section we discuss the data, definitions and annotation study. We follow with results, a discussion section and a conclusion.

## Methods

In this section we will discuss the data and definitions for the annotation task. We also describe the annotation study.

### Data

The data to be annotated consists of two corpora of web pages created by the author: IBS70 and MMED100. The MMED100 corpus was created for annotation purposes by randomly selecting ten documents from each of the ten queries used to create a larger corpus (MMED1000). Both corpora are described below.

At this time neither corpus is publicly available. However they can be provided on request to researchers and it is anticipated that they will be made publicly available once a viable standard is established for the annotations.

### IBS70 corpus

The IBS70 corpus was created as an exploratory corpus for use in system development. It was originally the top 50 Google hits for "irritable bowel syndrome" downloaded automatically though the Google API on July 1, 2004. The query was chosen to provide a range of quality and types of pages which one would expect to see more generally in the medical domain on the web: patient information from both traditional and alternative sources, support groups, medical articles, commercial pages from drug companies and quacks.

During system development we determined that it would be useful to have additional pages at both ends of the reliability spectrum, possibly to use as seeds for clustering. On September 15, 2004, twenty documents were added to the corpus to create the IBS70. Ten highly reliable documents were added based on web searches to find documents judged as meeting the standards of Evidence Based Medicine. Ten documents judged unreliable were added by taking the first ten relevant “Sponsored Links” resulting from a Google search on “irritable bowel syndrome”.

### MMED100 corpus

The MMED1000 corpus was created on November 5th and 8th, 2004 by automatically downloading from Google the top 100 search results for each of the following 10 queries:

*Adrenoleukodystrophy*, *Alzheimer's*, *Endometriosis*, *Fibromyalgia*, *Obesity*, *Pancreatic cancer*, *Colloidal Silver*, *Irritable Bowel Syndrome*, *Late Lyme Disease*, and *Lower Back Pain*

The queries were chosen to provide a broad range of what might be typical queries for health consumers on the web and the types of pages that would result from these queries.

Colloidal Silver was chosen in the hopes of providing a sufficient number of pages of questionable reliability. Adrenoleukodystrophy, Pancreatic Cancer, Alzheimer’s and Obesity were chosen because there is general agreement in the medical community that these are diseases or health issues, and on diagnostic techniques. They also cover a spectrum of occurrence rates, with Adrenoleukodystrophy being relatively rare and Obesity being relatively common. The other five queries were chosen because there is less agreement in both the medical community and the general population about the existence, frequency, severity and treatment of these conditions.

### Definitions

We classify consumer medical web pages along two axes: reliability and type. The primary task of the automated system is to classify pages based on their reliability (quality or trustworthiness of the information they contain). The secondary task, to classify pages based on their type (e.g. commercial, patient leaflet, link), emerged from the hypothesis that different types of pages may need to be treated differently to classify them based on their reliability. For example, if the primary purpose of a page is to provide links to information, determining the reliability of the page may require determining the reliability of the pages to which it links. However, for the current state of the automated system and in the current annotation study, annotators are provided only the given web page and are not allowed to follow links, so their reliability determination was made based on the apparent balance and objectivity of the links on the page.

The following definitions are taken from the instructions provided to the annotators with some minor editing for clarity. For both tasks, only one tag was allowed, so annotators were instructed to consider the main purpose or intent of the page.

### Reliability

Reliability of web pages is annotated based on a four level reliability scale, with a fifth tag option for pages where there is insufficient information to make a determination.

#### Probably reliable

The information on these pages appears to be complete and correct, meeting the standards of Evidence-Based Medicine where appropriate. Information is presented in a balanced and objective manner, with the full range of options discussed (where appropriate). The page and author appear reputable, with no obvious conflicts of interest. The appropriate disclaimers, policies, and contact information are present. Where appropriate, sources are cited. An example of a page in this category would be a patient leaflet from a reputable source that adheres to the standards of Evidence-Based Medicine.

#### Possibly reliable

The information on the page is generally good and without obvious false or outdated statements, but may not be sufficiently complete and balanced or may not conform to evidence-based standards. An example of a page in this category would be a patient leaflet that contains only a brief description of diagnostic procedures or suggests a treatment option that is generally accepted, but not supported by evidence.

#### Possibly unreliable

These pages may contain some reliable information, but either have some that is outdated, false or misleading, or the information is sufficiently unbalanced so as to be somewhat misleading. For example a practitioner commercial page, which has valid information about an illness, but only discusses the preferred treatment offered by the practitioner.

#### Probably unreliable

These pages contain false or misleading information, or present an unbalanced or biased viewpoint on the topic. Examples of pages in this category would include: testimonials (unsupported viewpoints or opinions of a single individual) or pages that are clearly promoting and selling a single treatment option.

#### Unable to determine

This class is for pages where it is difficult or impossible to determine the reliability, generally because there is not enough information. For example, the page may be blank, only contain login information, or be the front page of a medical journal.

### Type of page

We found six types of pages that frequently appear in search results for queries in the consumer medical domain: Commercial, Patient Leaflet, Link, Medical Articles, Support, and Testimonials. There are also pages which are not relevant, or do not contain sufficient information to make a determination. Below we discuss each of these types. When a page seems to overlap categories the annotation is based on the primary purpose of the page.

#### Commercial

The primary purpose of these pages is to sell something. For example, pages about an ailment sponsored by a company, which sells a drug, treatment or equipment, to treat the condition. Given the desire to sell, these pages may present incomplete or unbalanced information (making them less likely to be reliable). Practitioner pages with no real (substantial) information, which are designed to get people to make an appointment, as opposed to patient leaflets (designed to supplement information that patients receive in the office or clinic), might also fall into this category.

#### Link

The primary purpose of these pages is to provide links to other pages or sites (external), which will provide information about a certain illness or medical condition. These links may or may not be annotated, and the degree of annotation may vary considerably. The reliability of these pages depends on the reliability of the pages they link to and annotations (if present). However, one can estimate the reliability of a page without following the links by considering the range and apparent objectivity of the links.

#### Patient leaflet, brochure, fact sheet or FAQ

The primary purpose of these pages is to provide information to patients about a specific illness or medical condition. Generally, these pages will be produced by a clinic, medical centre, physician, or government agency, etc. The primary purpose is to provide information. These pages will tend to have headings such as: symptoms, diagnosis, treatment, or prognosis. These headings can take the form of links to specific parts of the same page or to other pages on the same site (internal). The reliability of these pages is based on their content and determined by factors including Evidence-Based Medicine, completeness, and the presence of incorrect or outdated information. This class of pages needs to be distinguished from medical articles, especially in encyclopaedias, handbooks, or manuals (e.g. Merck Manual).

#### Medical article (practitioner or consumer)

The primary purpose of these pages is to discuss an aspect of a specific illness or medical condition, or a specific illness or medical condition. These can be divided into two main categories: articles aimed at consumers and articles aimed at health practitioners.

Articles aimed at health practitioners, particularly doctors, may be scientific research articles. The reliability of these pages is based on their content and determined by factors including Evidence Based Medicine, completeness, and the presence of incorrect or outdated information. Articles aimed at consumers may come from a variety of sources including mainstream and alternative media sources. Reliability is determined based on the content.

#### Support

The primary purpose of these pages is to provide support of sufferers (or their loved ones, or care-givers) of a particular illness or condition. The pages may contain information, similar to that found in a patient leaflet; links to other sites, similar to a links page; and testimonials. In addition they may contain facilities such as chat rooms, newsletters, and email lists. Activities may include lobbying for funding for research. These pages are generally created by individuals or non-profit organizations. For reliability, one may need to look at the agenda of the authors or group. It may be in their interest (politically) to overstate the problem or make things out to be worse then they are to secure increased funding or sympathy for their cause.

#### Testimonial

The primary purpose of these pages is to provide testimonial(s) of individuals about their experience with an illness, condition, or treatment. While individuals may be considered reliable when discussing their own personal experiences, these pages tend to be unreliable, because they are generally not objective or balanced. There is a tendency for readers to generalize from very specific information or experiences provided by the testimonial, which can be misleading.

#### Not relevant

These pages are blank or not relevant and include: login pages, conditions of use pages, and medical journal front pages.

### Annotation study

In order to start system development, a single annotator, X, who was involved with development of both the classifications and the system, tagged the IBS70 and MMED100. Then in Spring 2008 two senior undergraduate science majors (chemistry and biology), Y and Z, were hired for the annotation study. The annotation study consisted of two phases: training and testing. Each phase is described below.

### Training phase

The two student annotators, Y and Z, received copies of the draft annotation instructions. They each met individually with X to discuss the instructions and any questions they had.

For each of three training runs, ten randomly chosen web pages from the IBS70 corpus were posted on a private web site. The students annotated the pages for reliability and type and then met individually to discuss their annotations with X. As questions and issues arose, the instructions were amended to reflect clarifications. For example, Z needed additional instructions on the distinction between Link and Patient Leaflet pages; a previous separate category for FAQs was collapsed into the Patient Leaflet category.

After the first two training runs the “correct” tags were posted on the private web site and the students were allowed to refer to them for the rest of the study. While it was not explicitly stated that X’s tags were the gold standard, none of X’s tags changed as a result of the discussions and modifications to the instructions.

### Testing phase

Once the student annotators achieved reasonable levels of agreement (Cohen’s Kappa above 0.4) on each task, there was a three-part testing phase. The remaining 40 pages in the IBS70 corpus were randomly divided into two test corpora and finally the MMED100 corpus was annotated.

During the testing phase, one of the students, Z, seemed to annotate less carefully. (Possibly because the timing coincided with graduation and summer vacation.) For example, on the MMED100 corpus Z tagged 30% as N (unable to determine the reliability), compared to 12% for Y and 10% for X. Z was asked to go back and reconsider the web pages tagged as N. We report results with Z’s reconsidered tags here for completeness; further discussion will focus on agreement between X and Y.

## Results

We report inter-rater agreement using accuracy, Cohen’s Kappa statistic [9] for chance corrected agreement, and F-Measure [10]. We consider each annotation axis separately. Inter-coder agreement was moderate (0.77 accuracy, 0.62 F-measure, 0.49 Kappa) on the reliability axis and good (0.81 accuracy, 0.72 F-measure, 0.73 Kappa) along the type axis.

### Page reliability

After examining the results for the five reliability classes across all corpora, shown in Table 1, it was clear that the annotators were not able to make the more fine-grained distinctions between “probably” and “possibly” for either the reliable or unreliable classes, given the current instructions and timeline. The classes were then collapsed to three: reliable, unreliable and unable-to-determine, and the results are shown in Table 2. We estimate a baseline distribution of the categories reliable, unreliable, and unable-to-determine based on an average of the tags across all training and test sets: 68% reliable; 19% unreliable; 13% unable-to-determine.

**Table 1:** Inter-rater agreement on page reliability for each corpus: 5-classes.

Accuracy/ Kappa	Reliability: 5 classes		
**Set\Raters**	**X-Y**	**X-Z**	**Y-Z**

**IBS train**	0.47 / 0.30	0.33 / 0.12	0.40 / 0.19
**IBS test**	0.33 / 0.11	0.40 / 0.25	0.43 / 0.28
**MMED100**	0.51 / 0.32	0.35 / 0.12	0.38 / 0.14

**Table 2:** Inter-rater agreement on page reliability for each corpus: 3-classes.

Accuracy/ Kappa	Reliability: 3 classes		
**Set\Raters**	**X-Y**	**X-Z**	**Y-Z**

**IBS train**	0.70 / 0.44	0.60 / 0.25	0.67 / 0.33
**IBS test**	0.70 / 0.43	0.65 / 0.42	0.75 / 0.59
**MMED100**	0.77 / 0.49	0.66 / 0.30	0.62 / 0.22

The results in Table 2 for X-Y show improved agreement after training and consistent moderate agreement on the test corpora based on the Kappa statistic. Accuracy (percent agreement) for X-Y is 70% for both IBS testing and training and 77% for the MMED100.

Further analysis of Z’s reliability tags showed a bias toward the “unreliable” tag. For example, in the MMED100 corpus, Z tagged 28% as unreliable, compared to 19% and 17% for X and Y, respectively.

Hripcsak and Rothschild [10] suggest use of the F-measure (harmonic average of precision – equivalent to positive predictive value - and recall – equivalent to sensitivity - commonly used in Information Retrieval) to calculate inter-rater agreement in the absence of a gold standard. In Table 3 we report the average F-measure between each pair of raters and the F-measure by class (the highest average was 0.62 for X-Y). A higher F-measure indicates better agreement, so these results show that the “Unable-to-Determine” class is the most difficult to agree on, followed by the “Unreliable” class.

**Table 3:** F-measure by class on page reliability for MMED100 corpus: 3-classes.

MMED100 F-Measure	Reliability: 3 classes		
**Class\Raters**	**X-Y**	**X-Z**	**Y-Z**

**Reliable**	0.87	0.78	0.76
**Unreliable**	0.55	0.46	0.36
**Unable to Det.**	0.45	0.22	0.30
**Average**	**0.62**	**0.49**	**0.47**

In order to look for patterns of agreement between the raters we looked at agreement by query in the MMED100 corpus. In Table 4 we show the agreement for X and Y by query (accuracy ranges from 40% to 100%). Although it appears that some queries were easier to annotate than others, since there are only 10 pages per query, the sample may be too small to draw definite conclusions.

**Table 4:** Inter-rater reliability agreement by query for X-Y for MMED100 corpus.

Query	Accuracy	Kappa
**Endometriosis**	1.00	1.00
**Pancreatic Cancer**	1.00	1.00
**Late Lyme**	1.00	1.00
**Adrenoleukodystrophy**	0.80	0.41
**Obesity**	0.80	0.66
**Alzheimer’s**	0.70	-0.15
**Fibromyalgia**	0.70	0.44
**Lower Back Pain**	0.70	-0.15
**Colloidal Silver**	0.60	0.13
**Irritable Bowel Syndrome**	0.40	-0.05

### Page type

The dominant page types are patient leaflets, link, commercial and medical article. The baseline distribution based on averages across the training and test sets is: 39% patient leaflets; 15% link; 18% commercial; and 13% medical article. The other three classes support, testimonial, and not relevant, making up only 15% of the pages in the corpus.

Table 5 shows the results for accuracy and the Kappa statistic on the seven type classes across all the corpora. The highest accuracy was 0.81 for X-Y on the MMED100 corpus. Collapsing categories for the type annotation task did not appreciably increase Kappa scores (X-Y Kappa was 0.74 compared to 0.73 on the MMED100 corpus when the patient leaflets and medical article classes were collapsed), so it seems preferable to keep the original classes.

**Table 5:** Inter-rater agreement for page type annotation for each corpus.

Accuracy/Kappa	Type		
**Set\Raters**	**X-Y**	**X-Z**	**Y-Z**

**IBS train**	0.57 / 0.42	0.83 / 0.78	0.47 / 0.28
**IBS test**	0.73 / 0.64	0.65 / 0.55	0.73 / 0.64
**MMED100**	0.81 / 0.73	0.48 / 0.29	0.50 / 0.31

Again we see improved agreement from training to testing for annotators X and Y as distinctions between classes were clarified (for example, between link and patient leaflets, and between patient leaflets and medical articles).

We also computed F-measure by type for the MMED100 corpus, shown in Table 6, the average across types for X-Y was 0.72. Of the three most common types of pages (patient leaflet, link, commercial), the link type was the most difficult for X-Y to agree on.

**Table 6:** F-measure by class for page type for MMED100 corpus.

MMED100 F-Measure	Type		
**Class\Raters**	**X-Y**	**X-Z**	**Y-Z**

**Patient Leaflet**	0.89	0.59	0.63
**Link**	0.63	0.48	0.44
**Commercial**	0.73	0.32	0.41
**Support**	0.77	0.22	0.25
**Testimonial**	0.50	0.00	0.80
**Medical Article**	0.67	0.59	0.46
**Not Relevant**	0.86	0.14	0.12
**Average**	**0.72**	**0.34**	**0.44**

We further analyzed the page type annotations by query for raters X and Y with the results shown in Table 7 (accuracy ranges from 70% to 90%). We found a negative correlation between the variance of the types resulting from a query to the Kappa statistic of agreement for the query (r = -0.62).

**Table 7:** Inter-rater page type agreement by query for X-Y for MMED100 corpus.

Query	Accuracy	Kappa
**Endometriosis**	0.90	0.85
**Fibromyalgia**	0.90	0.85
**Alzheimer’s**	0.80	0.75
**Irritable Bowel Syndrome**	0.80	0.73
**Obesity**	0.80	0.70
**Pancreatic Cancer**	0.80	0.63
**Colloidal Silver**	0.80	0.63
**Adrenoleukodystrophy**	0.8	0.512
**Lower Back Pain**	0.8	0.512
**Late Lyme**	0.7	0.483

## Discussion

The results of this annotation study show that the definitions and instructions we have developed for estimating the reliability and page type of consumer medical web pages can produce reasonable to good human agreement. The study also raises some questions, which we will discuss below, as well as avenues for future work.

Given the proliferation of inaccurate, false and misleading medical information on the Web, it will be increasingly important to provide heath consumers with tools to enable them to find reliable health information. There do exist online questionnaires (e.g. [11], [12]) and semi-automated systems have been explored (e.g. [13]). However, our goal is to develop a completely automated system using supervised machine learning algorithms [4], which requires annotated examples to learn from. This study is a step toward validating our definitions and creating additional annotated data to train our system.

Annotation studies have been discussed and conducted in the computational linguistics community for a variety of annotation tasks, including subjectivity (e.g. [14]) and opinion (e.g. [15]). Artstein and Poesio [16] surveyed inter-coder agreement in computational linguistics, including Cohen’s Kappa. The current study methodology and the use of the Kappa statistic are based on the standards developed in the computational linguistics community.

We are using an iterative development process for system development including data annotation. We are currently in the relatively early stages of development and working a coarse-grained level. For purposes of validating definitions, given limited resources, we used annotators with science and health backgrounds, who are not medical experts. They were capable of identifying most of the page attributes described in the instructions. However, as development progresses and more precise annotations are needed we anticipate conducting a new study with “expert” annotators – having a stronger medical background – and additional training. An intermediate option might be to ask annotators to use existing web tools (e.g. [3], [11], [12]) to assess the page quality. This would allow us to compare the results of annotating with web tools to annotating with our instructions.

In future studies agreement on reliability does need to be improved through a combination of refining and clarifying definitions, using annotators with greater expertise, and possibly with additional training for the annotators. Agreement on the page type is good, however it is still to be determined if noise levels are low enough and sufficiently random to be used successfully in supervised learning. The page-type annotation task is easier than the reliability task and requires less expertise of the annotators.

To pursue our goal of creating a “gold standard” for training machine learning algorithms to do automatic classification a number of approaches could be used, including: the production of bias-corrected tags as described by Weibe et al. [14]; systematically assess whether the noise introduced by moderate agreement levels will create problems for machine learning with this data [17].

## Conclusions

There is a demonstrated need to provide tools to health care consumers to automatically filter web pages by the reliability, quality, or trustworthiness of the medical information the pages contain. We have shown promising results in this study that appropriate classes of pages can be developed. These classes can be used by human annotators to annotate web pages with reasonable to good agreement.

Thus we have laid a foundation for future annotation studies to create a gold standard data set of consumer medical web pages. The corpora in this study are currently being used to create an automated system to estimate the reliability of medical web pages. Preliminary results of the system are comparable on this task to work done on similar tasks with similar methodology [4].

## List of abbreviations used

FAQ: frequently asked questions

## Competing interests

The author declares no competing interests.
